# Natalizumab Therapy Modulates miR-155, miR-26a and Proinflammatory Cytokine Expression in MS Patients

**DOI:** 10.1371/journal.pone.0157153

**Published:** 2016-06-16

**Authors:** Giuseppe Mameli, Giannina Arru, Elisa Caggiu, Magdalena Niegowska, Stefania Leoni, Giordano Madeddu, Sergio Babudieri, Gian Pietro Sechi, Leonardo A. Sechi

**Affiliations:** 1 Department of Medical Sciences, University of Sassari, Sassari, Italy; 2 Department of Experimental Medicine, University of Sassari, Sassari, Italy; Ospedale Pediatrico Bambino Gesu', ITALY

## Abstract

MicroRNAs fine-tune the regulation of Th1/Th17 lymphocyte subsets in multiple sclerosis. We investigated the expression of miRNAs (previously associated with mycobacterial and viral infections) in MS patients and healthy donors (HD) following 6 months natalizumab therapy. In addition, Th1/Th17 cytokines and the presence of anti-EBNA1/VCA IgG in MS patients with different pattern of miRNA expression have been evaluated. MiR-155, miR-26a, miR-132, miR-146a and Th1/Th17 cytokines expression was detected by RT-real time PCR; moreover anti-EBNA1 and VCA IgG titres were measured by ELISA. We observed an up-regulation of miR-155 (p value = 0.009) and miR-132 (p value = 0.04) in MS patients compared to HD. In MS patients, IL-17a (p = 0.037), IFN γ (p = 0.012) and TNFα (p = 0.015) but not IL-6 were over-expressed compared to HD. Two different miRNAs patterns associated to the expression of different cytokines were observed in the MS cohort. Moreover, a down-regulation of miR-155 and miR-26a was seen in MS patients during and after natalizumab therapy. MS patients that over-expressed miR-155 showed a higher EBNA1 IgG titer than MS patients with high levels of miR-26a. In conclusions the expression of particular miRNAs modulates the pro-inflammatory cytokine expression and the humoral response against EBV and this expression is natalizumab regulated.

## Introduction

Multiple sclerosis (MS) is an heterogeneous disorder of the central nervous system (CNS) that begins as an inflammatory autoimmune disease mediated by auto-reactive lymphocytes, followed by microglial activation and chronic degeneration with consequent brain and spinal cord myelin destruction. The aetiology of MS disease is still unknown although different infectious agents may trigger the pathogenic cascade [1,2]. MS is characterized by an immune-mediated inflammatory response, the up-regulation of Th1 and Th-17 cells and the presence of related cytokines in peripheral blood mononuclear cells (PBMCs) and cerebrospinal fluid; active lesions of multiple sclerosis patients during relapsing phases have been also demonstrated [3]. MicroRNAs (miRNAs) are a class of small non-coding RNAs (19–25 nucleotides) which regulate gene expression post-transcriptionally by binding to mRNA targets; this results in degradation or transcriptional repression of the targeted mRNA with a consequent decrease of encoded proteins [4–6]. Previous studies have reported involvement of different miRNAs in regulation of Th1 and Th17 differentiation from naïve CD4+ T cells in association with pathogenesis of autoimmune diseases such as multiple sclerosis and rheumatoid arthritis^.^ [7,8]. Therefore, the aim of the present study was to evaluate the expression levels of previously selected miR-155, miR-132, miR-146a and miR-26a in PBMCs of MS patients compared to healthy donors (HD). Previous studies displayed them implicated in immune-mediated inflammatory response and cytokines levels in MS [9–12] We also monitored the expression of these miRNAs before and after 6-month infusion of the humanized anti-α4 integrin monoclonal antibody natalizumab, because they have been described as good candidates for disease biomarkers in natalizumab-treated patients. [13–15]. Another aim of miRNA analysis was to find a possible relation between their expression and immune response against EBNA-1 and VCA IgG levels, two important EBV markers MS related in patients before and after natalizumab therapy.

## Materials and Methods

### Patients

Twenty four sardinian MS patients with clinically defined RRMS [16,17] (F/M = 3.8; mean age 35.6±8.5), referred to the Centre for MS Diagnosis and Treatment, Dept. of Clinical and Experimental Medicine (Neurology), University Hospital of Sassari, were enrolled in the study, Table 1. Blood samples were collected immediately before the first Natalizumab infusion (T0) and after six months (T6). Current infections and treatment with intravenous steroids within one month preceding the study were exclusion criteria. Twenty-four sex-matched healthy donors (HD) at the Blood Transfusion Centre of Sassari were used as control subjects (F/M = 3.7, mean age 37±9.5). Immediately after collection, peripheral blood mononuclear cells (PBMCs) were isolated from 10ml of blood by density gradient centrifugation on Ficoll-Paque Plus, (GE Healthcare Bioscience, Sweden), washed twice in phosphate-buffered saline (PBS), counted and stored at -80°C with RNA later (Sigma) until further use.

**Table 1:** Clinical characteristics of patients treated with natalizumab (groups T0, T6).

	n	mean	SD	Range (min-max)
Sex (female/males)	19/5			
Age		35.6	8.5	19–61
EDSS		3.83	1.62	1–6.5
Pre-medication	15 interferon beta			
	8 glatirameracetate			
	1 azathioprine			
Years of disease		8.63	8	2–41
Disease form (RRMS)	RR			

### Ethics statement

The study was approved by the University of Sassari and ASL No. 1 Bioethical Committee. The patients and the volunteers gave written informed consent.

### MiRNAs cDNA synthesis and real-time PCR

Purification of total RNA containing miRNA from PBMCs was performed using miRNeasy Mini kit (Qiagen,) according to the manufacturer’s recommendations. Quality of extracted RNA was determined according to 260/280 absorbance ratio, measured by Nano Drop spectrometer (Thermo Scientific, USA). 500 ng/RNA were used in reverse-transcription reaction. cDNA synthesis for miR-155, miR-132, miR-146a and miR-26a was fulfilled using a miSCript II RT Kit (Qiagen) according to the manufacturer’s leaflet. MiRNAs quantification was performed with Custom miScript miRNA PCR Array. The cDNA amplification was carried out by using standard protocols with a Biorad I cycler instrument (Biorad, USA). miRNAs data analysis was performed using the ΔΔCT method by Qiagen miRNA detection software and final data were normalized for small nuclear RNA, miRTC (median Ct = 24.86 ± 0.614) PPC (median Ct = 21.27± 0.302), RNU6-6P (median Ct = 23.34 ± 0.116), SNORD68 (median Ct = 22.54± 0.211) expression levels as endogenous controls [18,19,20,21]

### Heat Maps

We performed heat maps on using GeneGlobe Data Analysis Center (Qiagen). The heat map provides a visualization of the fold changes in expression between the selected groups for every gene in the array in the context of the array layout. The table provides the fold regulation data used for the map as well as the Comments associated with each one. The colour of the square denotes the relative up- or down-regulation of the miRNA in that sample. In addition, it produces dendrograms for the rows and columns, which are computed using hierarchical clustering. The ordering of the rows and columns is the one most compatible with the dendrograms.

### CDNA and Real-time PCR for cytokines

Total RNAs were extracted from 100.000 cells, by Trizol (sigma) and retrotranscribed, as described [22]. selective amplification of the IL-17a, IFN γ, TNFα and IL-6 transcripts was obtained by real time PCR assay, with specific primers [23,24]. Data have been expressed according to the 2^-ΔCt^ method, conforming to the. Parallel RNA samples were also exposed to PCR amplification without the RT step, to detect contaminant DNA. For each sample, the Ct (cycle threshold) value of the gene of interest (GI) was normalized by comparison to the Ct of the glyceraldehyde-3-phosphate dehydrogenase (GAPDH) invariate housekeeping gene. The data have been expressed according to the 2-ΔCt Method [22].

### ELISA IgG EBNA1 and VCA

Serum EBV-specific IgG antibodies were detected by commercially available Chemiluminescence Immunoassays (CLIA), following the manufacturer’s instructions (LIAISON® VCA IgG, and EBNA-1 IgG, DiaSorin S.p.A. Vercelli, Italy).

### Statistics

Statistical analysis was performed using GraphPad Prism 6.0 software (San Diego, CA, USA). The p values are calculated based on a Student t-test of the replicate 2^ (- Delta Ct) values for each gene in both compared groups and p values less than 0.05 are significant. Comparisons 2-ΔCt value of cytokines expression between different groups were tested using the chi-squared or Fisher exact test.

## Results

### Upregulation of miR-155 and miR-132 in multiple sclerosis patients

**Fig 1:**
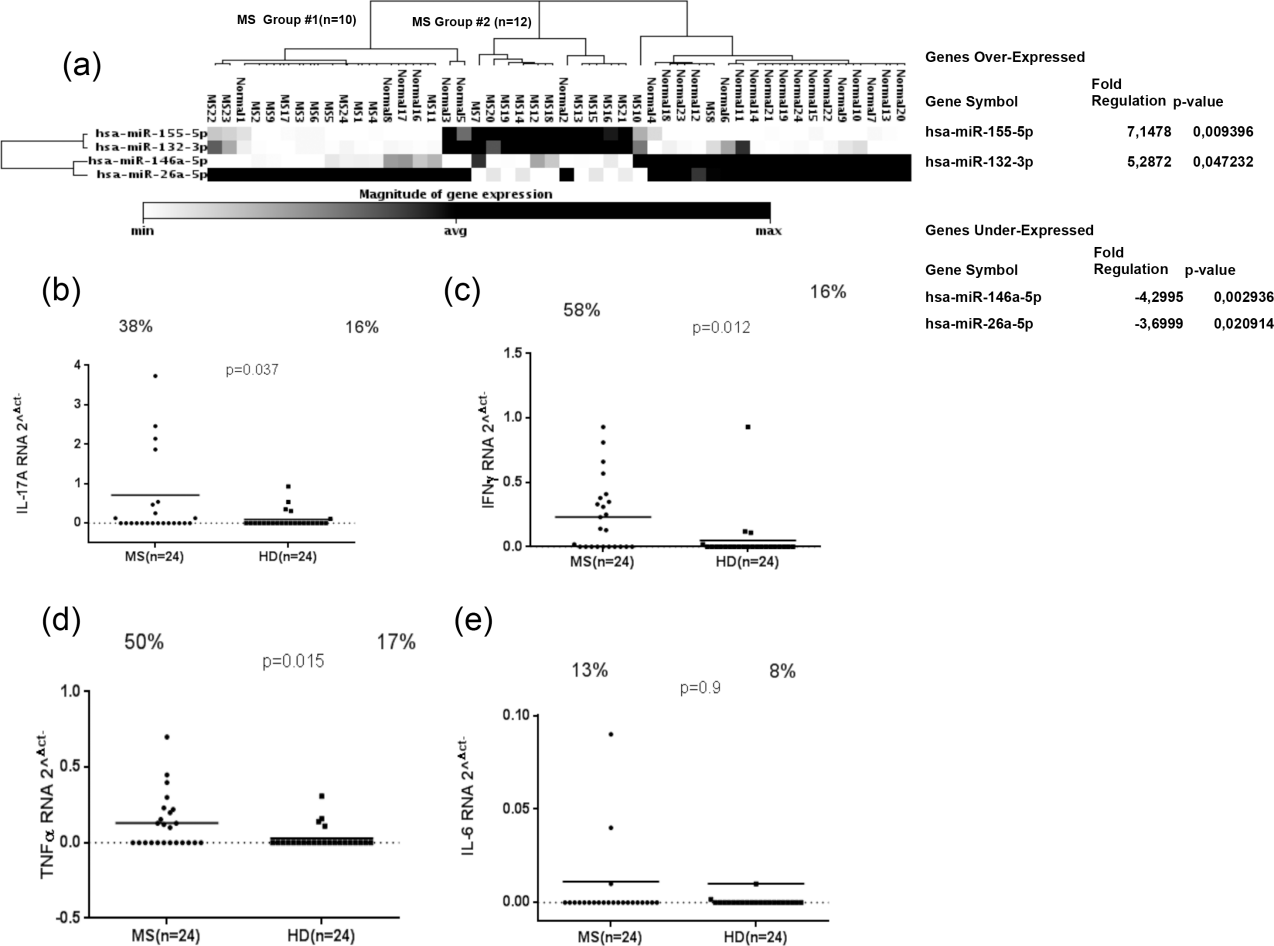
Magnitude of microRNAs expression: miR-155, miR-132, miR-146a and miR-26a expression in twenty-four MS patients and HDs (a). IL-17a, IFN γ, TNFα and IL-6 RNA expression in twenty-four MS and HD samples (b; c; d; e). The horizontal black bars represent the mean value, while percentages of positive samples are indicated on the top of each distribution. Cut-off values for positivity, calculated by ROC analysis, are indicated by dashed lines.

Relevant microRNAs, including miR-155, miR-132, miR-146a and miR-26a, were investigated in PBMC samples from MS and HD. MicroRNAs data analysis showed that miR-155 (fold change = 7.15; P = 0.009) and miR-132 (fold change = 5.28; P = 0.047) were up-regulated in 24 MS patients compared to HD, whereas miR-146a (fold change = -4.29; P = 0.0032) and miR-26a (fold change = -3.69; P = 0.02) were down-regulated, (Fig 1A). We then sought to examine IL-17a, IFN γ, TNFα and IL-6 expression in MS patients and in HDs. Our results showed that IL-17a gene expression was significantly higher in MS than in HD, (Fig 1B) (P = 0.037) and also 38% MS samples were positive against 16% positives for HD. As well, IFN γ and TNFα genes were significantly higher expressed in MS in comparison to HDs, (Fig 1C and 1D). The percentage of samples positive to IFN γ was statistically significant for MS and HD samples (58% against 16%, respectively) (p = 0.012; Fig 1C); the same trend was observed for TNFα (50% of MS samples in comparison to 17% HD) (p = 0.015; Fig 1D). Conversely, IL-6 mRNA expression didn’t show any difference between MS and HD (p = 0.9; Fig 1E).

### Different regulation of miR-155, miR-132, miR-146a and miR-26a in multiple sclerosis patients and correlation with cytokines expression

**Fig 2:**
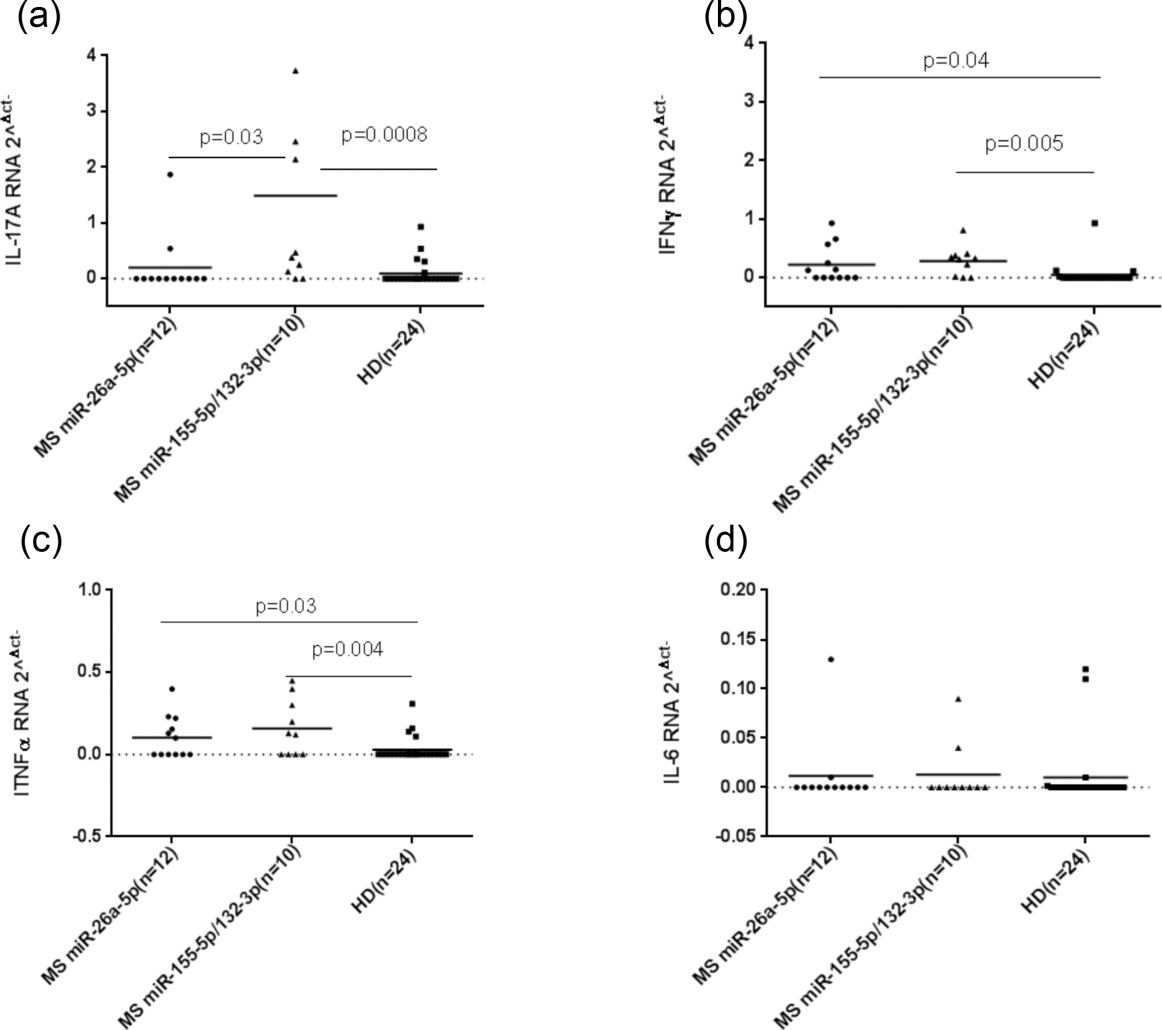
Real-time RT-PCR of IL-17a, IFN γ, TNFα and IL-6 relative to twelve MS patients with high levels of miR-155 and miR-132, and ten MS patients with high levels of miR-26a (a; b; c; d). The horizontal black bars represent the mean values.

A different pattern of miRNAs expression in MS patients was observed. In ten out of twenty-four MS patients, both miR-155 and miR-132 were up-regulated (MS group #1), whereas up-regulation of only miR-26a was registered in other twelve MS patients (MS group #2) as shown in (Fig 1A). In order to evaluate the association between the regulation of different miRNAs with cytokines expression in PBMCs of MS patients and HDs, we measured expression levels of IL-17a, IFN γ, TNFα and IL-6 mRNA in the MS group that overexpressed miR-155/miR-132 and in the MS patients with demonstrated up-regulation of miR-26a compared to HD. IL-17a was expressed at significant levels only in the MS group characterized by a higher level of both miR-155/miR-132 in comparison to the MS group with the increased expression of miR-26a and HD (Fig 2A). Whereas both Th1 cytokines IFN γ and TNFα were significantly expressed in these two MS groups when compared to HD (Fig 2B and 2C). We did not detect any difference in the expression of IL-6 mRNA in MS and HDs (Fig 2D).

### Th1/Th17 cytokines regulation in multiple sclerosis patients before and after 6 months of natalizumab therapy

**Fig 3:**
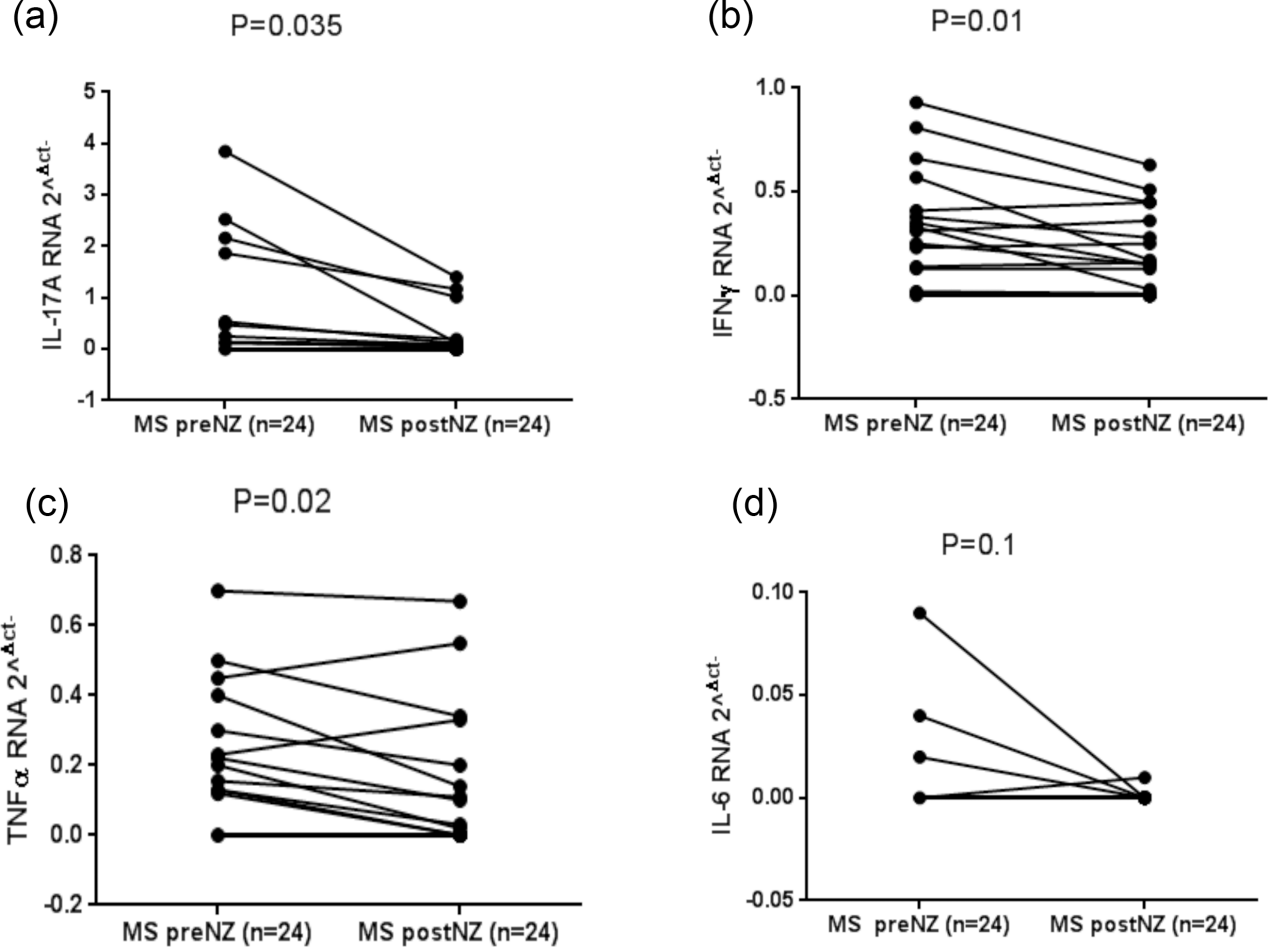
RNA cytokine fragment relative amounts of IL-17a, IFN γ, TNFα and IL-6 in twenty-four MS patients before and after six months of natalizumab treatment (a; b; c; d).

Different types of cytokines expression in PBMCs of MS patients before and after natalizumab treatment were detected by real-time RT-PCR. IL-17a gene expression was significantly decreased after 6 months of natalizumab therapy as shown in (Fig 3A), (P = 0.035). A similar trend was observed for IFN γ and TNFα gene expression in MS patients with a significant down-regulation after 6 months of therapy (P = 0.01; P = 0.02), (Fig 3B and 3C). On the other hand, IL-6 mRNA expression was not modified during natalizumab treatment as depicted in (Fig 3D).

### Different regulation of miR-155, miR-132, miR-146a and miR-26a in multiple sclerosis patients before and after 6 months of natalizumab therapy

**Fig 4:**
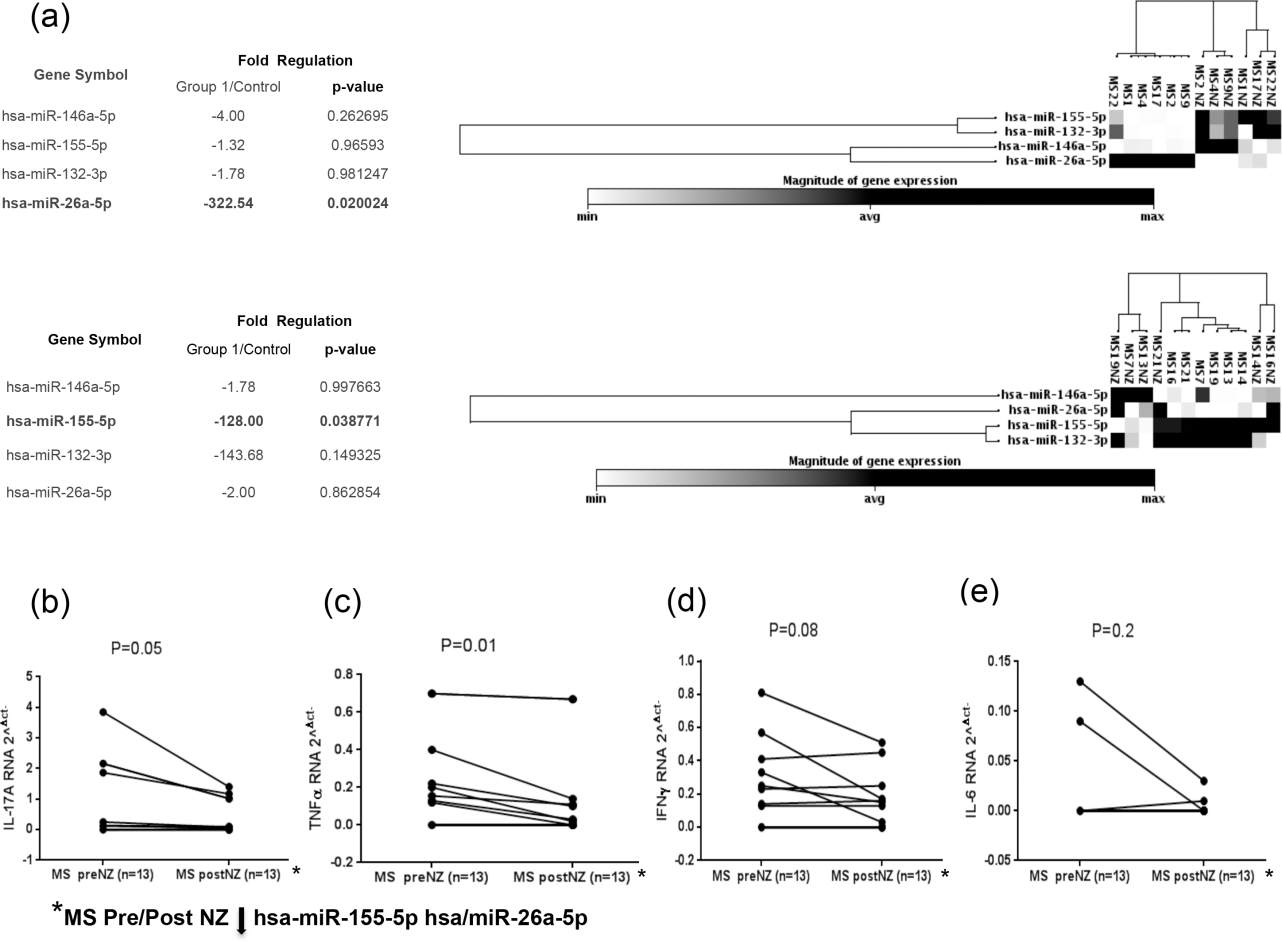
Magnitude of microRNAs: miR-155, miR-132, miR-146a and miR-26a expression in twelve MS patients before and after 6 months of natalizumab therapy (a). IL-17a, IFN γ, TNFα and IL-6 RNA expression in thirteen MS patients with miR-155 and miR-26a levels decreasing from the condition before therapy to the period following 6 months of natalizumab treatment (b; c; d; e).

MicroRNAs analysis in MS patients before and after 6 months of natalizumab therapy showed a significant down-regulation of miR-155 and miR-26a miRNAs (Fig 4A). Subsequently, we evaluated the cytokines mRNA expression in PBMCs of 13 MS patients with concomitant down-regulation of miR-155 and miR-26a in pre- or post-therapy individuals. Both cytokines IL-17a and TNFα were significantly under-expressed in MS patients that showed a significant down-regulation of miR-155 and miR-26a after drug treatment (Fig 4B and 4C), whereas IFN γ and IL-6 mRNA levels were not significantly decreased in the same MS group (Fig 4D and 4E)].

### Detection of EBNA1 and VCA IgG levels in multiple sclerosis patients before and after 6 months of natalizumab therapy and possible association with miRNA regulation

**Fig 5:**
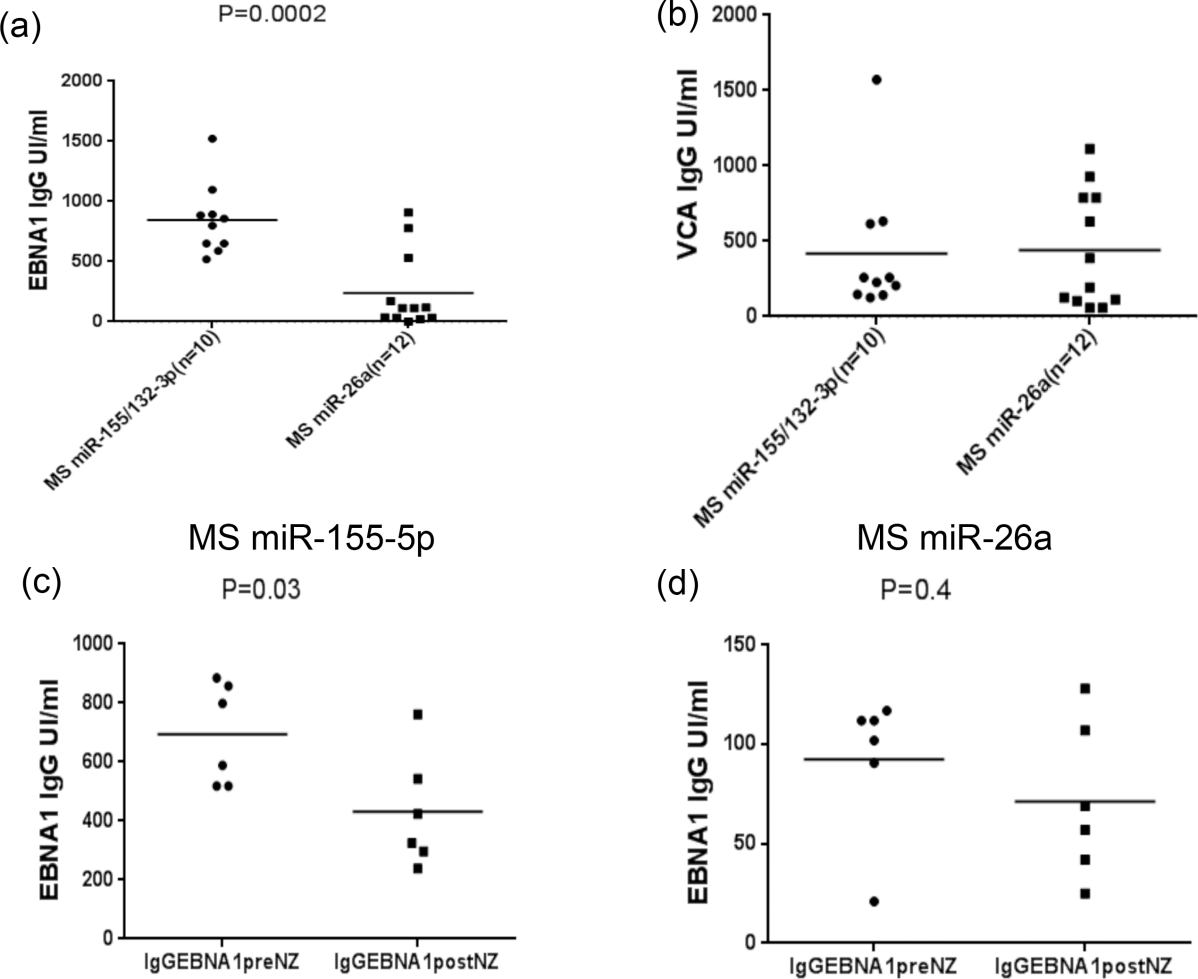
Antibody titers measured by Kit ELISA, EBNA1 and VCA specific IgG antibodies detected in twelve MS patients with high levels of miR-155 and miR-132, and ten MS patients with increased miR-26a expression (a; b). Detection of EBNA1 and VCA IgG levels in six MS patients with down-regulation of both miR-155 and miR-26a before and after 6 months of natalizumab treatment (c; d). The horizontal black bars represent the mean values.

Concerning EBNA1 and VCA IgG levels, no statistically significant difference was observed in the antibody level during natalizumab exposure when student’s t-test was applied at samples between T0 and T6 (data not shown). Conversely, in MS samples that showed higher miR-155 pattern, we found a significantly high levels of anti-EBNA1 IgGs but not anti-VCA IgGs in comparison to MS individuals that showed an over-expression of miR-26a, (Fig 5A and 5B). In order to evaluate the association between the expression of different miRNAs with anti-EBNA1 and VCA IgGs levels during natalizumab treatment, we observed lower levels of EBNA1 IgG titers only in the miR-155 down-regulated MS group but not in the miR-26a MS group after 6 months of therapy, (Fig 5C and 5D).

## Discussion

Dysregulation of miRNAs has been associated with several autoimmune diseases [23,25,26]. Different types of miRNAs are regulated in blood and brain lesions of multiple sclerosis (MS) patients [27,28]. MiRNAs can be detected in blood and are related to different types of Th1/Th17 cytokines expression before and during natalizumab therapy [13,15,23,24,29]. Interestingly, miRNAs are stable in blood samples and this strongly suggests that PBMCs miRNA can be used as a potential clinical biomarker [30]. We focused our attention on miRNA-146a, miRNA-155 and microRNA-132 because they are probably implicated in pro-inflammatory processes cytokines mediated in MS while miR-26a is a potential marker for diagnosis of relapse and remission phases. The results show that a higher miR-155 and miR-132 expression in PBMC was associated to MS patients compared to healthy donors, but the results also showed two different patterns of miRNAs expression within MS patients: the first group over-expressed miR-155 and miR-132 in concert with up-regulation of the associated Th1/Th17 cytokine types such as IL-17a, IFN γ, TNFα but not IL-6, whereas the second MS group characterized by high levels of miR-26a, over-expressed only Th1 cytokine types such as IFN γ and TNFα. We didn’t find any correlation between microRNAs pattern and clinical manifestation in our cohort of MS patients. In the second part of the study we checked miRNAs and cytokines regulations in MS patients before and after 6 months of natalizumab treatment. A down-regulation of miR-155 and miR-26a after 6 months of therapy and a consistent down-regulation of Th1/Th17 cytokines was observed. We reported a higher serum prevalence of EBNA-1 IgG in MS patients with high levels of miRNA-155. This data was confirmed by other groups that showed the ability of EBV to induce the expression of miR-155 [31], during latent infection mediated by EBNA1 and LMP, indeed EBV is able to modulate the expression of specific cellular miRNAs, such as miR-155 and miR-146 [31]. Previous work did not show that natalizumab therapy was able to modify the immune response against EBNA1 in MS patients [32,33]. On the other hand, our results show that natalizumab treatment is able to decrease the immune response against EBNA1 only in MS patients with higher expression of miR-155, probably because of a strong interaction between EBV and miRNA155 [34,35,36] To sum up, the results obtained are potentially promising and suggest the presence of altered regulation patterns of miR-155, miR-132, miR-146a in MS patients along with different miR-26a expression before and after natalizumab therapy, that in turn might promote a different Th1/Th17 cytokine response.
